# A Versatile Click-Compatible Monolignol Probe to Study Lignin Deposition in Plant Cell Walls

**DOI:** 10.1371/journal.pone.0121334

**Published:** 2015-04-17

**Authors:** Jyotsna L. Pandey, Bo Wang, Brett G. Diehl, Tom L. Richard, Gong Chen, Charles T. Anderson

**Affiliations:** 1 Department of Agricultural and Biological Engineering, The Pennsylvania State University, University Park, Pennsylvania, United States of America; 2 Department of Chemistry, The Pennsylvania State University, University Park, Pennsylvania, United States of America; 3 Department of Biology, The Pennsylvania State University, University Park, Pennsylvania, United States of America; 4 Center for Lignocellulose Structure and Formation, The Pennsylvania State University, University Park, Pennsylvania, United States of America; Iowa State University, UNITED STATES

## Abstract

Lignin plays important structural and functional roles in plants by forming a hydrophobic matrix in secondary cell walls that enhances mechanical strength and resists microbial decay. While the importance of the lignin matrix is well documented and the biosynthetic pathways for monolignols are known, the process by which lignin precursors or monolignols are transported and polymerized to form this matrix remains a subject of considerable debate. In this study, we have synthesized and tested an analog of coniferyl alcohol that has been modified to contain an ethynyl group at the C-3 position. This modification enables fluorescent tagging and imaging of this molecule after its incorporation into plant tissue by click chemistry-assisted covalent labeling with a fluorescent azide dye, and confers a distinct Raman signature that could be used for Raman imaging. We found that this monolignol analog is incorporated into *in vitro*-polymerized dehydrogenation polymer (DHP) lignin and into root epidermal cell walls of 4-day-old *Arabidopsis* seedlings. Incorporation of the analog in stem sections of 6-week-old *Arabidopsis thaliana* plants and labeling with an Alexa-594 azide dye revealed the precise locations of new lignin polymerization. Results from this study indicate that this molecule can provide high-resolution localization of lignification during plant cell wall maturation and lignin matrix assembly.

## Introduction

Lignin is a phenolic polymer that is abundant in the secondary cell walls, middle lamellae and cell corners of mature plant cells [1]. It is hypothesized to function mainly as a structural component of secondary walls, aiding in upright plant growth [1–3]. It is hydrophobic and is thought to function as a waterproofing agent in some cell types, causing water to be excluded from secondary walls and facilitating transpirational water flow through plant vascular tissue [1,4]. Additionally, lignin helps plants resist microbial attack by diminishing the efficiency of secondary wall degradation [5,6], which also means that it is a major contributor to the recalcitrance of biomass to depolymerization during processing for the production of biofuels [7–9]. Lignin polymerization appears to begin in the cell corners and middle lamellae of lignifying plant tissues, before spreading through the cell walls [1,10]. Lignin concentration is usually higher in the middle lamellae and cell corners than in the secondary cell wall [10–12], but since the secondary walls occupy larger volumes they contain most of the lignin mass [1]. Lignin is derived mainly from three monolignol substituents, coniferyl alcohol, sinapyl alcohol and *p-*coumaryl alcohol, although other phenolic monomers can also be incorporated into the polymer [1,4,13–15].

Monolignols are synthesized in the cytoplasm of plant cells from phenylalanine by a series of enzymatic modifications including deamination, hydroxylation, *O*-methylation, and the conversion of the carboxyl group to an alcohol. The mechanism by which these monolignols are then transported to the extracellular space before polymerization is a subject of debate [16,17]. Some recent studies suggest that the likely mechanism of transport is through ATP-binding cassette-like transport proteins, also called ABC transporters [18–22]. Once transported across the plasma membrane, the monolignols polymerize via radical coupling reactions that are thought to be initiated by laccases and peroxidases [1,4,7,13,20]. The chemical, rather than enzymatic, nature of lignin polymerization makes the process quite flexible [4,7], and modified monolignols that are endogenously synthesized or exogenously supplied can be incorporated readily into the polymer [23,24]. Lignin can also interact, either non-covalently or via covalent linkages, with other cell wall constituents such as cellulose, hemicelluloses, and pectins [25–27]. However, the nanoscale details of lignin distribution and the kinetics of its polymerization over short timescales remain unclear. Recent advances in our ability to probe lignin structure and distribution include the ability to detect it using spectroscopic methods such as NMR and Raman [28–35], and imaging methods such as fluorescence microscopy [10,36–38]. In one recent study focusing on the latter technique, the authors linked two different fluorophores to coniferyl alcohol to generate a pair of monolignol derivatives and were able to observe the incorporation of these derivatives into *in vitro-* and *in vivo-*polymerized lignins [23,24]. However, it is possible that the large sizes of these fluorophores interfere with their incorporation into lignin, preventing them from accurately reflecting the precise location and kinetics of natural lignin polymerization in plant tissues.

**Fig 1:**
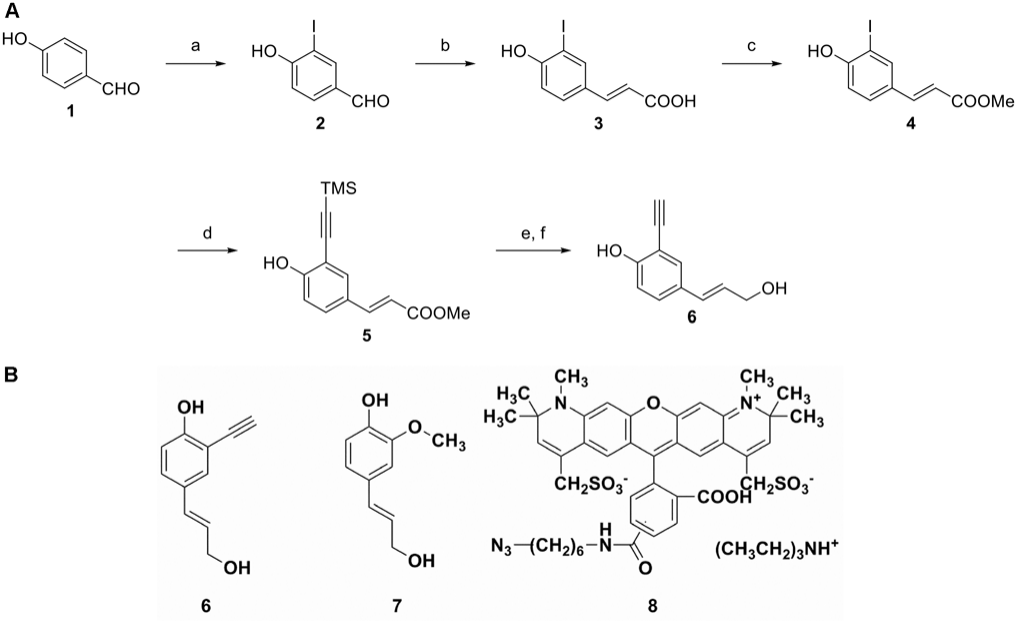
Synthesis of 3-ethynyl *p-*coumaryl alcohol (3-EPC), 6. (A) Reagents and conditions: a) Iodine monochloride, acetic acid, dichloromethane, 24 hours, 25°C; b) malonic acid, pyridine, piperidine, 90°C, 3.5 hours; c) methanol, sulfuric acid, reflux, overnight; d) trimethylsilyl acetylene, Bis(triphenylphosphine)palladium(II) chloride, cuprous iodide, triethylamine-tetrahydrofuran, 50°C, overnight; e) Diisobutylaluminium hydride (DIBAL-H), dichloromethane, -78°C, 3 hours; f) tetrabutylammonium fluoride, tetrahydrofuran, 25°C, 1 hour. (B) Chemical structures of monolignols and fluorophore. (E)-2-ethynyl-4-(3-hydroxyprop-1-enyl)phenol or 3-ethynyl *p-*coumaryl alcohol (3-EPC), **6**, the monolignol probe developed in this paper; coniferyl alcohol (CA), **7**, a natural lignin monomer; and Alexa 594-azide, **8**, for fluorescence imaging.

In the past decade, copper-catalyzed click chemistry has emerged as a useful approach for bioorthogonal metabolic labeling of living cells. In this approach, a metabolite modified with an alkynyl or azido group is added to the cells, which incorporate it into their structure. After incorporation, the modified metabolite can be detected by performing a copper-catalyzed click reaction that covalently links a detection probe, often a fluorophore, to the modified metabolite. This protocol has been used to label carbohydrates and nucleic acids in a variety of organisms, including bacteria, animals, and plants [39–46], allowing for tracing of the dynamics of incorporation and modification of polymers containing the modified metabolite. Due to its chemical mode of polymerization, lignin should be amenable to the incorporation of a click-compatible monolignol analog, allowing for the efficient detection of lignification *in vitro* and in plant tissues. In this study we designed and synthesized a new coniferyl alcohol analog, (E)-2-ethynyl-4-(3-hydroxyprop-1-enyl) phenol, or 3-ethynyl *p-*coumaryl alcohol (3-EPC), **6** (Fig 1A), and tested its incorporation in plant tissues. Very recently, incorporation and labeling of click-compatible monolignols into plant tissues was demonstrated by Tobimatsu *et al*., where the designed monolignols were modified at the γ–O-site instead of the 3’-C position [47]. Another study by Bukowski *et al*., demonstrated the incorporation and click-labeling of coniferyl alcohol modified at the 3’-C position to include a propargyl group instead of an ethynyl group [48]. In this study, we use nuclear magnetic resonance (NMR) spectroscopy to show that 3-EPC can be incorporated into lignin dehydrogenation polymer (DHP, an *in vitro* lignin model polymer), and is thus compatible with lignin polymerization. We also demonstrate the incorporation of 3-EPC into specific locations in actively lignifying plant tissues, as detected using fluorescence labeling and imaging. Sites of 3-EPC incorporation can be differentiated from previously existing lignin, which is detectable by its autofluorescence at a different excitation wavelength. Together, these results provide information about the chemical flexibility of lignin polymerization and the process by which plant cell walls become lignified.

## Materials and Methods

### Reagents and chemicals

Coniferyl alcohol (Fig 1B) and Horseradish peroxidase (type II, 150–250 units/mg) were purchased from Sigma. Alexa 594-azide (Fig 1B), Murashige and Skoog salts, Shandon Cryomatrix resin, and monohydrate (2-(N-Morpholino)ethanesulfonic acid) (MES) were purchased from Life Technologies, Caisson Labs, Thermo Scientific, and Research Organics, respectively. All commercial materials for the chemical synthesis of 3-EPC were used as received unless otherwise noted. Flash chromatography was performed using 230–400 mesh SilicaFlash 60 silica gel (Silicycle Inc.). *p*-hydroxybenzaldehyde (99%, Acros), iodine monochloride (1 M solution in dichloromethane, Aldrich), malonic acid (99%, Alfa Aesar), trimethylsilyl acetylene (98%, Matrix Scientific), Bis(triphenylphosphine)palladium(II) chloride (99%, Aldrich), Diisobutylaluminium hydride (1 M solution in hexane, Acros), and tetrabutylammonium fluoride (1 M solution in tetrahydrofuran, Acros) were used for the chemical synthesis of 3-EPC. Other commercial chemicals, including solvents, were of reagent grade, purchased from Sigma-Aldrich, and used without further purification.

### Synthesis of 4-hydroxy-3-iodobenzaldehyde (2)

A solution of iodine monochloride in dichloromethane (1 M, 180 mL) was added dropwise to a solution of *p-*hydroxybenzaldehyde, **1** (11.2 g, 92.1 mmol) and acetic acid (27 mL) in dichloromethane (200 mL) at room temperature. The resulting solution was stirred at room temperature for 24 h followed by washing with saturated sodium thiosulfate aqueous solution and then saturated sodium chloride aqueous solution (brine). Flash chromatography on silica gel using dichloromethane as eluent gave 10.7 g of product **2** as a pale yellow solid with 47% yield. ^1^H NMR (DMSO, 360 MHz, ppm): δ 7.02–7.05 (d, J = 8.6 Hz, 1H), 7.76–7.78 (d, J = 8.3 Hz, 1H), 8.23 (s, 1H), 9.75 (s, 1H), 11.57 (br s, 1H); ^13^C NMR (DMSO, 90 MHz, ppm) δ 85.61, 115.57, 130.57, 131.70, 141.80, 162.70, 190.58; HRMS: calculated for C_7_H_6_IO_2_ [M+H+]: 248.8412; found: 248.9413.

### Synthesis of (E)-3-(4-hydroxy-3-iodophenyl)acrylic acid (3)

A solution of 4-hydroxy-3-iodobenzaldehyde, **2** (10.7 g, 43 mmol), malonic acid (6.6 g, 63 mmol), pyridine (22 mL) and piperidine (0.22 mL) in toluene (25 mL) was heated at 90°C for 3.5 h. After being cooled to room temperature, a solution of potassium carbonate (13 g of potassium carbonate dissolved in 65 mL of water) was added. After stirring vigorously for 15 min, the resulting solution was transferred to a separatory funnel, and the aqueous layer was separated and transferred into a 250 mL flask and cooled using an ice-water bath. Hydrochloric acid was carefully added to the aqueous layer to adjust the pH to 1–2. The precipitate was collected by filtration and dried, giving 11 g of compound **3** with 88% yield. ^1^H NMR (DMSO, 360 MHz, ppm): δ 6.33–6.37 (d, J = 15.8 Hz, 1H), 6.89–6.91 (d, J = 8.3 Hz, 1H), 7.44–7.48 (d, J = 15.8 Hz, 1H), 7.56–7.58 (d, J = 8.3 Hz, 1H), 8.02 (s, 1H), 11.0 (br s, 1H), 12.2 (br s, 1H); ^13^C NMR (DMSO, 90 MHz, ppm) δ 85.64, 115.53, 117.14, 127.96, 130.06, 139.54, 143.09, 159.00, 168.22; HRMS: calculated for C_9_H_8_IO_3_ [M+H+]: 290.9518; found: 290.9518.

### Synthesis of (E)-methyl 3-(4-hydroxy-3-iodophenyl)acrylate (4)

To a solution of compound **3** (10 g, 34 mmol) in methanol (200 mL), sulfuric acid (1 mL) was carefully added at room temperature. The resulting solution was refluxed overnight. After cooling to room temperature, the solution was concentrated under vacuum to remove methanol. The residue was dissolved in 200 mL of ethyl acetate, and washed with water and then brine. Concentration under vacuum gave 9.5 g of product **4** as a pale yellow solid with 90% yield. ^1^H NMR (DMSO, 360 MHz, ppm): δ 3.70 (s, 3H), 6.44–6.48 (d, J = 15.8 Hz, 1H), 6.89–6.92 (d, J = 8.3 Hz, 1H), 7.51–7.55 (d, J = 16.2 Hz, 1H), 7.58–7.60 (d, J = 8.3 Hz, 1H), 8.06 (s, 1H), 10.95 (s, 1H); ^13^C NMR (DMSO, 90 MHz, ppm) δ 51.81, 85.68, 115.51, 115.68, 127.74, 130.28, 139.71, 143.68, 159.24, 167.31; HRMS: calculated for C_10_H_10_IO_3_ [M+H+]: 304.9675; found: 304.9678.

### Synthesis of (E)-methyl 3-(4-hydroxy-3-((trimethylsilyl)ethynyl)phenyl)acrylate (5)

To a solution of compound **4** (5 g, 16.5 mmol), bis(triphenylphosphine)palladium(II) chloride (116 mg, 0.165 mmol), cuprous iodide (63 mg, 0.33 mmol) in triethylamine (32 mL) and tetrahydrofuran (THF) (53 mL) at room temperature, trimethylsilyl acetylene (3.5 mL, 24.75 mmol) was added dropwise. The reaction mixture was heated at 50°C overnight. After completion, the reaction was diluted with ethyl acetate (100 mL), and washed with water and then brine. Flash column chromatography on silica gel using hexanes and ethyl acetate (10:1) as eluent gave 3.6 g of product **5** as a yellowish solid with 80% yield. ^1^H NMR (DMSO, 360 MHz, ppm): δ 0.23 (s, 9H), 3.70 (s, 3H), 6.46–6.51 (d, J = 15.8 Hz, 1H), 6.91–6.93 (d, J = 8.6 Hz, 1H), 7.53–7.57 (d, J = 16.2 Hz, 1H), 7.59–7.61 (d, J = 8.6 Hz, 1H), 7.68 (s, 1H), 10.63 (br s, 1H); ^13^C NMR (DMSO, 90 MHz, ppm) δ 0.50, 51.79, 98.20, 102.11, 110.74, 115.66, 116.45, 125.74, 130.93, 134.46, 144.20, 161.20, 167.40; HRMS: calculated for C_15_H_19_O_3_Si [M+H+]: 275.1103; found: 275.1100.

### Synthesis of (E)-2-ethynyl-4-(3-hydroxyprop-1-enyl)phenol or 3-ethynyl *p-*coumaryl alcohol (3-EPC) (6)

To a dry ice-acetone cooled solution of compound **5** (1.4 g, 5.1 mmol) in dichloromethane (50 mL), diisobutylaluminium hydride or DIBAL-H (1M in hexane, 16 mL, 16 mmol) was added dropwise. The reaction was kept at -78°C for 3 h until thin-layer chromatography (TLC) showed a complete consumption of compound **5**. After being warmed to room temperature, the reaction was quenched by dilute hydrochloric acid (1M, 10 mL). The organic layer was separated and washed thoroughly with 1 M hydrochloric acid to dissolve the floccule. The organic layer was then dried by anhydrous sodium sulfate, and concentrated under vacuum. The residue was used in the next step without further purification.

To a solution of the residue in THF (20 mL), tetrabutylammonium fluoride solution (1 M in THF, 8 mL, 8 mmol) was added at room temperature. After completion of the reaction indicated by TLC, the reaction was diluted with ethyl acetate and washed with water and then brine. Flash column chromatography on silica gel using hexane and acetone (2:1) as eluent gave 670 mg of product **6** as a yellow syrup with 75% yield. The product must be stored in a solution of acetone at -80°C to avoid decomposition.^1^H NMR (CDCl_3_, 300 MHz, ppm): δ 3.46 (s, 1H), 4.28–4.30 (d, J = 5.4 Hz, 2H), 6.18–6.25 (m, 1H), 6.45–6.50 (d, J = 15.9 Hz, 1H), 6.88–6.91 (d, J = 8.7 Hz, 1H), 7.27–7.29 (m, 2H), 7.38–7.39 (d, J = 1.5 Hz, 1H); ^13^C NMR (CDCl_3_, 75 MHz, ppm) δ 64.05, 78.76, 84.51, 109.00, 115.66, 127.39, 129.34, 129.64, 130.39, 130.72, 157.43; HRMS: calculated for C_11_H_11_O_2_ [M+H+]: 175.0759; found: 175.0760.

### Synthesis of dehydrogenation polymers (DHPs)

Lignin DHP was prepared in a similar manner to that previously reported [49]. A solution of 200 mg monolignols (50 mg 3-EPC + 150 mg CA for 25% 3-EPC DHP; 200 mg 3-EPC for 100% 3-EPC DHP; or 200 mg CA for G-DHP) in 1 mL of acetone was dissolved in 200 mL sodium phosphate buffer (0.01 M, pH 6.5). Four milligrams of horseradish peroxidase enzyme (HRP) was added to each solution to catalyze polymerization. A 200 mL solution of 0.025% hydrogen peroxide was prepared in sodium phosphate buffer (0.01 M, pH 6.5) and slowly combined with the monomeric alcohol-HRP solution into another flask containing 1 mg of HRP dissolved in 2 mL of sodium phosphate buffer (0.01 M, pH 6.5) at a rate of approximately 3 ml/h for 67 h, using a peristaltic pump. The reaction mixture was stirred for an additional 24 h to complete the polymerization. The precipitate was collected by centrifugation (10,000 *g*, 30 min, 4°C). The DHP precipitates were thoroughly washed 4X with 40 mL distilled water using high speed centrifugation (10,000 *g*, 30 min, 4°C) to ensure removal of any nonpolymerized monomers. The precipitates, comprised of non-soluble polymers, were then freeze-dried.

### Nuclear Magnetic Resonance (NMR) spectroscopy

NMR spectra of compounds **2**, **3**, **4**, **5** and **6** were recorded on Bruker AV-360 or Bruker CDPX-300 instruments (Billerica, MA, USA) and calibrated using residual solvent peaks as internal references. Solvents used for the NMR of small molecules included dimethyl sulfoxide (DMSO) for compounds **2** through **5** and deuterated chloroform for 3-EPC, **6**. Multiplicities are recorded as: s = singlet, d = doublet, t = triplet, dd = doublet of doublets, m = multiplet, q = quartet. High resolution ESI mass experiments were operated on a Waters LCT Premier instrument (Milford, MA, USA).

NMR spectra of the DHPs were collected in 4:1 DMSO-d6:pyridine-d5 because it is a preferred solvent for NMR of lignin DHP, milled wood lignin (MWL), and whole cell walls; using the same solvent system allows for accurate chemical shift comparisons. Spectra were acquired on a Bruker Biospin (Billerica, MA, USA) AVANCE 500 (500 MHz ^1^H resonance freq.) spectrometer fitted with a cryogenically-cooled probe, using a standard Bruker heteronuclear single quantum coherence (HSQC) pulse program (hsqcetgpsisp2.2, 56 scans with 32 mg of sample). Spectral processing was performed in Bruker’s Topspin 3.1 software using the central solvent peaks as internal reference (δH/δC: dimethyl sulfoxide (DMSO), 2.50/39.5 ppm) with chemical shifts reported in parts per million (ppm). Processing used typical matched Gaussian apodization in F2 (LB = -0.3, GB = 0.001), and squared cosine-bell and one level of linear prediction (32 coefficients) in F1 [50].

### Raman spectroscopy of DHPs

Raman spectra were collected using a Nicolet 8700 FT-Raman spectrometer. The DHP powders were excited using a diode-pumped 1064 nm Nd:YAG laser at 80 mW power and the signal was collected with a liquid nitrogen-cooled germanium detector. Fourier transform spectra were collected in the range 250–3500 cm^-1^ with data spacing of 1.928 cm^-1^ and were averaged from 500 scans. Raman spectra were baseline corrected and smoothed using OMINIC^TM^ spectrum analysis software (Thermo Scientific).

### Click labeling of DHPs and fluorescence measurements

Twenty milligrams of the freeze dried DHPs: G-DHP as a control, 25% 3-EPC DHP, or 100% 3-EPC DHP, were added to 4 ml of click labeling solution containing 1 mM ascorbic acid, 1 mM CuSO4 and 1 μM Alexa 594-azide (Life Technologies) in liquid MS medium (2.2 g/L Murashige and Skoog salts, 0.6 g/L MES, pH 5.6). Labeling was performed at 25°C in the dark with rocking for 1 h. The DHP suspension was then centrifuged at 20,124 g at 4°C for 20 min in an Eppendorf 5810R centrifuge with a swinging bucket rotor and the pellet was washed and pelleted 4X with milli-Q water using the same centrifugation conditions. The final pellet was freeze dried. A 5 mg/mL solution of the click labeled DHPs as well as unlabeled G-DHP were prepared in DMSO. The fluorescence values of these samples were analyzed with a FL3C-12 fluorometer (Horiba, New Jersey, USA). The system uses a 450W Xenon lamp as an excitation source with a single grating excitation monochromator, a T-format sampling module, a double grating emission monochromator, and a PMT detector. All slits and alignment optics were under instrument software control (FluorEssence with Origin, Horiba). Bandpass filters were set at 5 nm and spectrum integration time was 0.1 s. Five hundred microliter cuvettes were used for measurements. An excitation wavelength of 561 nm and an emission spectrum of 590–750 nm were used for fluorescence measurements.

### Incorporation, labeling, and imaging of *Arabidopsis* seedlings

*Arabidopsis thaliana* seedlings of the Col-0 ecotype were grown at 22°C under 24 h light for four days on plates containing solid Murashige and Skoog (MS) medium [2.2 g/L Murashige and Skoog salts + 0.6 g/L MES + 8 g/L agar-agar (Research Organics) + 10 g/L sucrose, pH 5.6]. After four days of growth the seedlings were transferred to microcentrifuge tubes with 1 mL liquid MS medium containing either 20 μM 3-EPC or 5 μM 3-EPC + 15 μM coniferyl alcohol (CA). Control seedlings were added to 1 mL liquid MS containing 20 μM CA. Seedlings were incubated in constant light at 22°C for 4 h. After incorporation, the seedlings were washed 4X with 1 mL liquid MS and transferred to microcentrifuge tubes with 1 mL of click-labeling solution containing 1 mM ascorbic acid, 1 mM CuSO_4_, and 0.1 μM Alexa 594-azide (Life Technologies) in liquid MS. Labeling was performed at 25°C in the dark with rocking for 1 h. Seedlings were washed 4X with 1 mL liquid MS before imaging on a Zeiss Cell Observer SD spinning disk fluorescence confocal microscope using a 561 nm excitation laser and a 617/73 emission filter with a 20X 0.5 NA air immersion objective or a 100X 1.4 NA oil immersion objective. Maximum projections of collected z series were generated using ImageJ, adjusting image brightness for all images to the same minimum and maximum gray scale values to enable comparisons of relative fluorescence intensities. Images were collected from a total of three replicate experiments with five seedlings imaged for each treatment.

### Incorporation, labeling, and imaging of *Arabidopsis* stem sections

Middle portions of six-week-old *Arabidopsis* Col-0 ecotype stems exhibiting secondary growth were cut into 8 mm pieces, embedded and frozen in Shandon Cryomatrix resin (Thermo Scientific), cryosectioned into 40 μm-thick sections using a Leica CM1950 cryostat, and placed in water. Sections were then transferred to 1 mL aqueous solutions of either 20 μM 3-EPC or 20 μM 3-EPC + 20 μM CA. Control sections were added to 1 mL of aqueous solutions of 20 μM CA. These sections were incubated at 22°C for 3 h. After incorporation, the sections were washed 4X with 1 mL water, transferred to 1 mL of click-labeling solution containing 1 mM ascorbic acid, 1 mM CuSO_4_, and 0.5 μM Alexa 594-azide in liquid MS medium and rocked at 25°C in the dark for 1 h. Sections were then washed 2X with 1 mL water, transferred to 1 mL of 96% ethanol, and rocked for 1 h to remove any unbound monomers or dyes before washing 4X with 1 mL water. Images were collected as above, with the addition of a 405 nm excitation laser and a 450/50 emission filter to image autofluorescence associated with lignin and a 63X 1.4 NA oil immersion objective. Maximum projections of z series were generated using ImageJ, adjusting image brightness for all images to the same minimum and maximum gray scale values to enable comparison of relative fluorescence intensities. Fluorescence intensities were quantified as raw integrated intensities per unit area in ImageJ after using a common threshold to select lignified regions. Separate threshold values were set for images acquired under the 405 nm and 561 nm channels. Images were collected from a total of three replicate experiments with three sections imaged for each treatment.

## Results and Discussion

### Synthesis and characterization of 3-EPC

To study the molecular details of lignification, we wanted to design a monolignol probe that, when incorporated into lignin, could be easily labeled and imaged using microscopy or be detected *in vivo* using spectroscopic tools. Click chemistry has been successfully implemented in labeling lignifying plant tissues [47], and using a similar but distinct strategy we designed 3-ethynyl *p-*coumaryl alcohol (3-EPC), a coniferyl alcohol analog that possesses a small, bioorthogonal terminal alkyne group at the 3’ position on the aromatic ring (Fig 1B), with a characteristic predicted Raman scattering signal. 3-EPC differs in structure from the clickable monolignols previously reported by Tobimatsu *et al*., where alkyne or azide modifications were introduced at the γ-O-position on the side chain [47]. The γ-O-position in monolignols has been previously modified [51–54], but has been reported to produce abnormal β–β-interunit linkages due to the lack of a γ-OH [55]. A recent study [56] has shown that the alkynyl C-H Raman signal can be considerably increased when the alkynyl group is directly linked to an aryl group, and we predicted that the alkynyl tag in 3-EPC should impart a strong Raman signal. Below, we report its characteristics and behavior when incorporated into lignin, during both *in vitro* and *in vivo* lignification.

As outlined in Fig 1A, the synthesis of 3-EPC started from the commercially available 4-hydroxybenzaldehyde **1**. The ortho-position of the starting compound **1** was first iodinated to give 3-iodo-4-hydroxybenzaldehyde, **2** [57]. Compound **2** was then converted into the corresponding substituted cinnamic acid, **3** through a Knoevenagel type condensation [58]. The acid **3** was then esterified by refluxing in methanol, giving the methyl ester, **4**. Palladium-catalyzed Sonogashira coupling [59] between the methyl ester **4** and trimethylethynylsilane gave the compound **5** in high yield. Finally the methyl ester group of compound **5** was reduced to an alcohol group by DIBAL-H, followed by removal of trimethylsilyl groups using TBAF to give the final product 3-EPC, **6**.

### Incorporation of 3-EPC during peroxidase-catalyzed dehydrogenative polymerization

To test whether 3-EPC would be oxidized by peroxidase and participate in radical coupling, and to determine whether and how this monolignol analog might be incorporated into *in vitro*-polymerized synthetic lignins, dehydrogenation polymers were prepared using 3-EPC and coniferyl alcohol in different proportions and chemically characterized.

**Fig 2:**
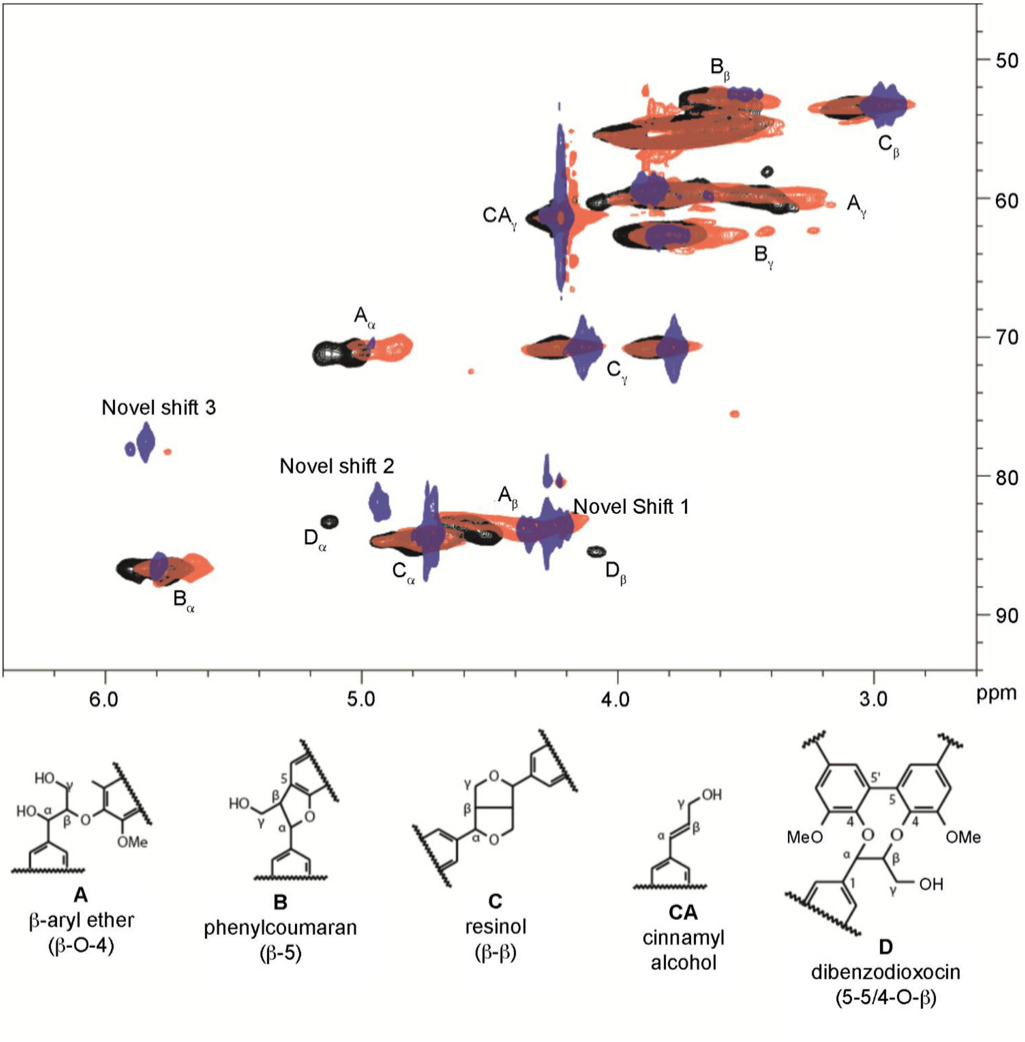
NMR spectra of the DHPs. The aliphatic regions of the HSQC 2D-NMR spectra of 100% coniferyl alcohol DHP (G-DHP, black), copolymer of 25% 3-EPC, **6** and coniferyl alcohol, **7** (red), and 100% 3-EPC, **6** DHP (blue).

**Fig 3:**
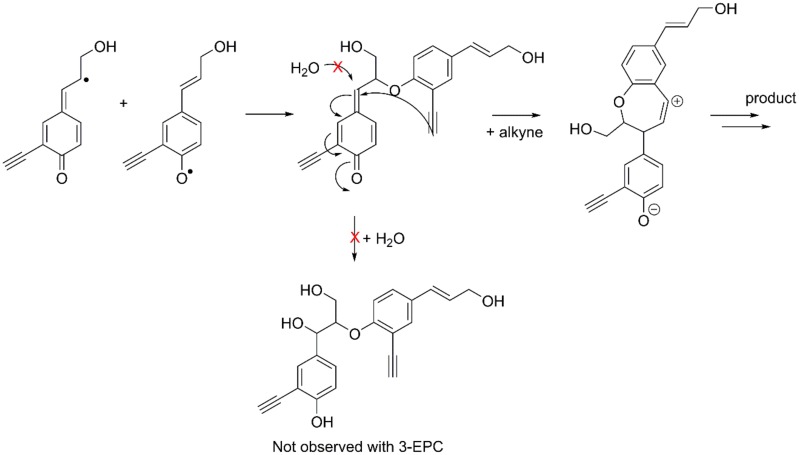
Proposed pathway for the formation of a novel β-ether linkage. For typical monolignols, external trapping of the β-ether quinone methide occurs via water addition (not experimentally observed with 3-EPC). The alternate pathway illustrates the proposed internal trapping of the quinone methide via the alkyne when 3-EPC is polymerized. The proposed carbocation would undergo additional reactions to yield an as-yet unidentified novel β-ether linkage type.

Heteronuclear single quantum coherence (HSQC) NMR spectra were collected for 100% coniferyl alcohol DHP (G-DHP, black), 25% 3-EPC DHP (red), and 100% 3-EPC DHP (blue) and are shown in Fig 2. The pure G-DHP contained shifts typical of G-DHPs. Interestingly, increasing the 3-EPC content dramatically decreased the shift near 5.0/72.0 ppm (A_α_), which was attributed to β-ether/α-hydroxyl linkages (i.e., “typical” β-O-4 linkages whose quinone methide intermediates were inter-molecularly quenched via reaction with water) [60]. In fact, when 3-EPC is the sole monolignol, β-ether/α-hydroxyl signals are essentially absent. Our interpretation of this result is that the alkyne group might react with the quinone methide intermediate in an intra-molecular fashion, as shown in Fig 3, to form a carbocation that undergoes additional transformation to yield an as-yet unidentified novel linkage type that has not been observed in natural lignins. This might happen so efficiently that β-ether/α-hydroxyl linkages do not form. Novel shift 1 (4.3/85.0 ppm) appears in the 25% 3-EPC sample and is prominent in the 100% 3-EPC sample at the expense of the shift at 4.6/85.0 ppm (A_β_, the β-O-4/α-OH β-position). This provides further evidence that β-ether/α-hydroxyl structures do not form, and novel shift 1 is likely attributable to a novel β-position. Novel shifts 2 (4.9/83.0 ppm) and 3 (5.8/78.0 ppm) remain unidentified, but one of these shifts is likely attributable to the α-position of the novel β-ether linkage type. Although the NMR data strongly suggest the formation of a new lignin inter-unit linkage type, most likely arising from participation of the alkyne in quinone methide trapping as in Fig 3, further work involving additional model lignin compounds will be necessary to determine the exact structure of this new linkage. Surprisingly, we did not detect unreacted alkyne functionality in the HSQC NMR spectra (Fig 3); additional proton and carbon NMR experiments (not shown) similarly failed to show alkyne shifts. As discussed above, some of the alkyne group may have been consumed via quinone methide trapping, or potentially via other side reactions.

**Fig 4:**
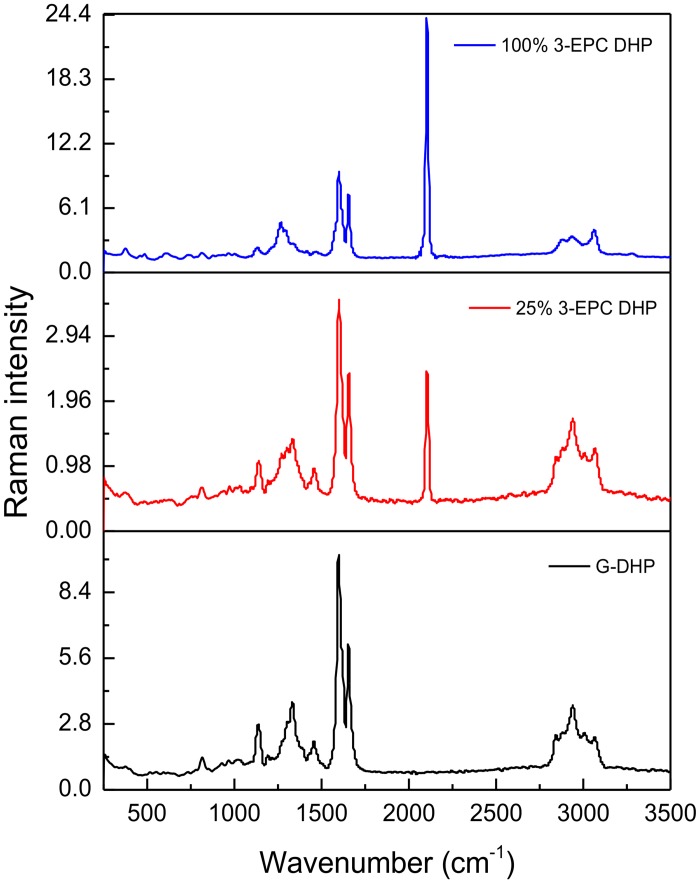
Raman spectra of DHPs. Spectra (1024 nm excitation, 500 scans, data spacing of 1.928 cm^-1^) of *in vitro*-synthesized DHP with 100% CA (black trace); DHP with 25% 3-EPC + 75% CA (red trace); and DHP with 100% 3-EPC (blue trace). The red and blue spectra show a characteristic terminal alkyne peak at 2100 cm^-1^.

Because 3-EPC contains a terminal alkyne group that should impart a characteristic Raman spectroscopic signal, Raman spectra for DHPs prepared with varying proportions of 3-EPC were collected and compared with G-DHP spectra. All of the DHPs showed characteristic lignin peaks at 1600 and 1650 cm^-1^ [31–33], but DHPs prepared with 3-EPC also showed an additional strong peak at 2100 cm^-1^, which corresponds to alkynyl stretching vibrations (Fig 4) [56]. This result indicated that at least some intact alkyne groups were present in the DHP and were detectable by Raman spectroscopy, but might have been below the detection threshold of NMR. Because this characteristic Raman peak does not correspond to any of the endogenous molecules present in cells [56,61], this result opens up the future possibility that Raman imaging could be used to detect the incorporation of 3-EPC into plant cell walls without additional labeling by reporter tags.

**Fig 5:**
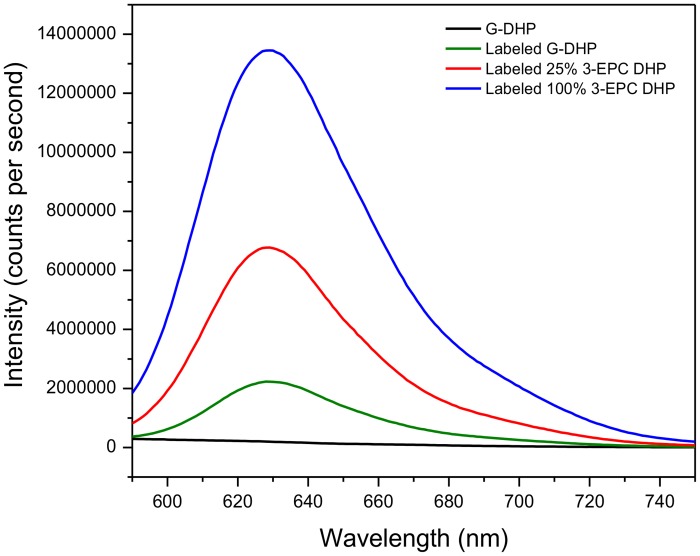
Fluorescence intensities (561 nm excitation) of click-labeled DHPs. 5 mg/ml solutions in DMSO of *in vitro*-synthesized DHPs incorporated with varying proportions of 3-EPC, **6** and click labeled with 1 μM Alexa 594-azide for 1 h in the dark at room temperature. Blue trace: click labeled 100% 3-EPC DHP; red trace: click labeled 25% 3-EPC + 75% CA DHP; green trace: click labeled 100% CA DHP or G-DHP; black trace: non-labeled G-DHP. The red and blue spectra show significantly higher fluorescence intensities compared to labeled and non-labeled G-DHP. Detectable, but low, fluorescence in click labeled G-DHP is possibly due to physical entanglement of the dye with the polymer.

To further test the hypothesis that the clickable monolignol analog 3-EPC was in fact incorporated into DHPs in a way that retained some intact alkynyl groups, we performed fluorescent labeling of DHPs prepared as described above using click chemistry. DHPs prepared with 25% or 100% 3-EPC were reacted with 1 μM Alexa 594-azide for 1 h at 25°C in the dark in the presence of Cu(I). Fluorescence measurements of these click-labeled DHP lignins (Fig 5) showed that the DHPs containing 3-EPC had much higher fluorescence intensities than G-DHP subjected to the same click-labeling conditions. G-DHP that was not click labeled was used as a negative control and showed negligible fluorescence at 561 nm excitation. Click labeled G-DHP, however, did show some fluorescence, even though it was much lower than that for 3-EPC-containing DHPs. A possible interpretation of this result is that a small amount of the Alexa 594-azide can become non-specifically entangled within G-DHP.

Even though alkyne peaks were not evident in the NMR data (Fig 2), we saw evidence for the presence of intact alkyne groups in DHPs polymerized with 3-EPC from their Raman spectra (Fig 4) as well as from click labeling followed by fluorescence measurements (Fig 5). We believe that these contrasting results might be due to the low sensitivity of NMR towards the small quantities of the alkyne group in the DHPs, whereas the sensitivities of Raman spectroscopy and fluorescence detection of labeled alkyne groups were higher.

### Incorporation of 3-EPC in *Arabidopsis* seedlings

**Fig 6:**
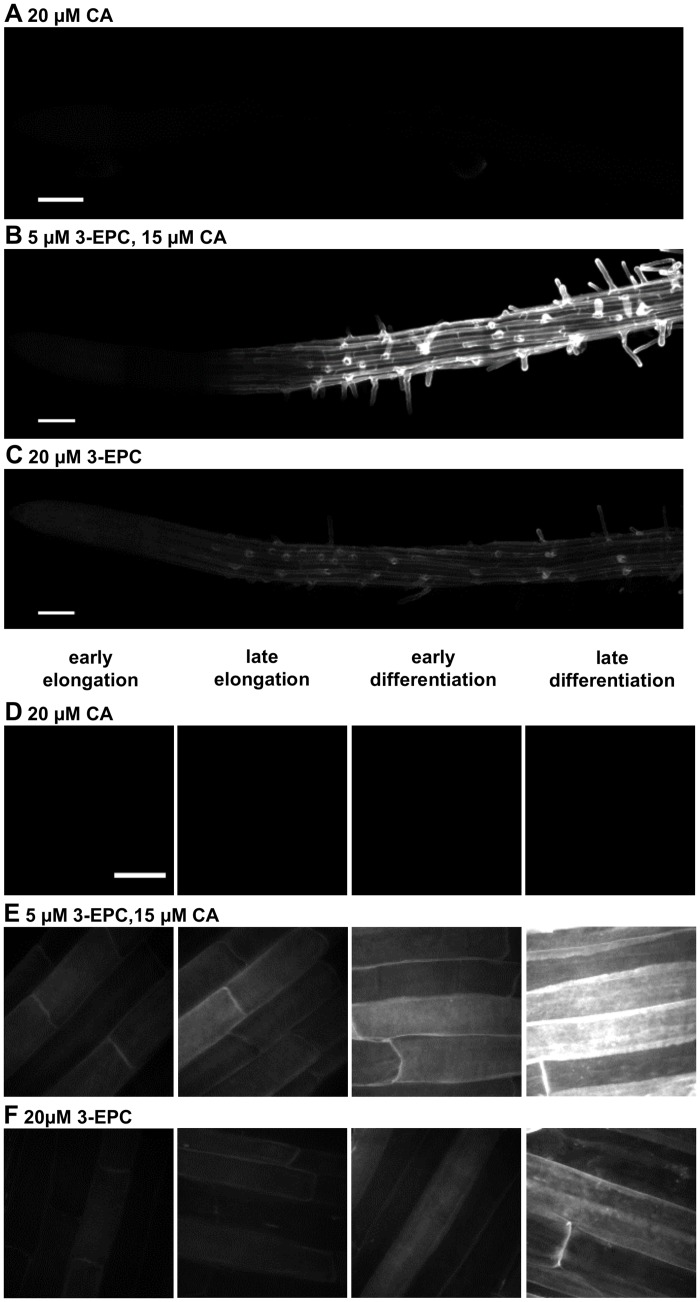
3-EPC incorporation in roots of 4-day-old *Arabidopsis* seedlings. (A, D) control seedlings treated with 20 μM CA for 4 h and labeled with Alexa-594 azide for 1 h. (B, E) seedlings treated with 5 μM 3-EPC and 15 μM CA for 4 h and labeled with Alexa-594 azide for 1 h. (C, F) seedlings treated with 20 μM 3-EPC for 4 h and labeled with Alexa-594 azide for 1 h. Images were collected with a spinning disk confocal microscope using a 561 nm laser at 10% power and 500 gain with an exposure time of 400 msec. (A-C) contrast-enhanced mosaics of contiguous images, starting at the root tip and going through the late differentiation zone, recorded using a 20X objective (Scale bar, 100 μm). (D-F) contrast-enhanced maximum intensity projections of z series recorded at the indicated root zones with a 100X oil-immersion objective (Scale bar, 20 μm).

After demonstrating that 3-EPC could be polymerized into a lignin-like polymer *in vitro*, we next investigated whether this monolignol analog could undergo polymerization in the cell walls of living plants. *Arabidopsis* Col-0 seedlings were incubated with 3-EPC in order to study its incorporation into root cell walls. Four-day-old seedlings were incubated with 3-EPC and/or CA in liquid MS medium for 4 h, reacted with Alexa 594-azide via a copper-catalyzed click reaction to fluorescently label any incorporated alkynyl groups, and imaged using fluorescence microscopy. Seedlings treated with 3-EPC showed significantly higher fluorescence in the root epidermis (Fig 6B and 6C, 6E and 6F) as compared to control seedlings that were treated with only coniferyl alcohol and reacted with Alexa 594-azide, which showed negligible fluorescence (Fig 6A and 6D). In seedlings treated with 3-EPC, fluorescence intensity increased from the root tip to the differentiation zone, with the highest intensities observed in the late differentiation zone (Fig 6E and 6F). Seedlings treated with a mixture of 5 μM 3-EPC and 15 μM CA to test whether the presence of a natural lignin precursor affects the incorporation of the synthetic monolignol showed significantly higher fluorescence (Fig 6B and 6E) as compared to seedlings treated with 20 μM 3-EPC (Fig 6C and 6F). Fluorescence quantification data for each of these treatments is shown in S7 Fig. The finding that the addition of CA resulted in higher incorporation of 3-EPC in the root epidermis suggests that either synergistic monolignol uptake might be occurring, or that 3-EPC self-reactivity in the absence of CA before or during its incorporation might result in the loss of free alkynyl groups.

These results suggest that 3-EPC is incorporated into the root epidermal cell walls of *Arabidopsis* seedlings and can be visualized by click chemistry-enabled fluorescent labeling of the monolignol tag. No fluorescence was observed in the vasculature of the roots, which might be due to low penetration of the Alexa dye into the root interior during the click labeling step of the experiment. The incorporation of 3-EPC in root epidermal cells of *Arabidopsis* seedlings was interesting because this tissue does not naturally contain large amounts of lignin. Incorporation of exogenous monolignols in non-lignifying tissues was observed in previous studies which used fluorescent monolignols [24] and click-compatible monolignols [48] in feeding experiments. This observation suggests that certain naturally non-lignifying tissues may possess the machinery required for lignification, which possibly results in incorporation of exogenously supplied monolignols.

### Incorporation of 3-EPC in *Arabidopsis* stem sections

**Fig 7:**
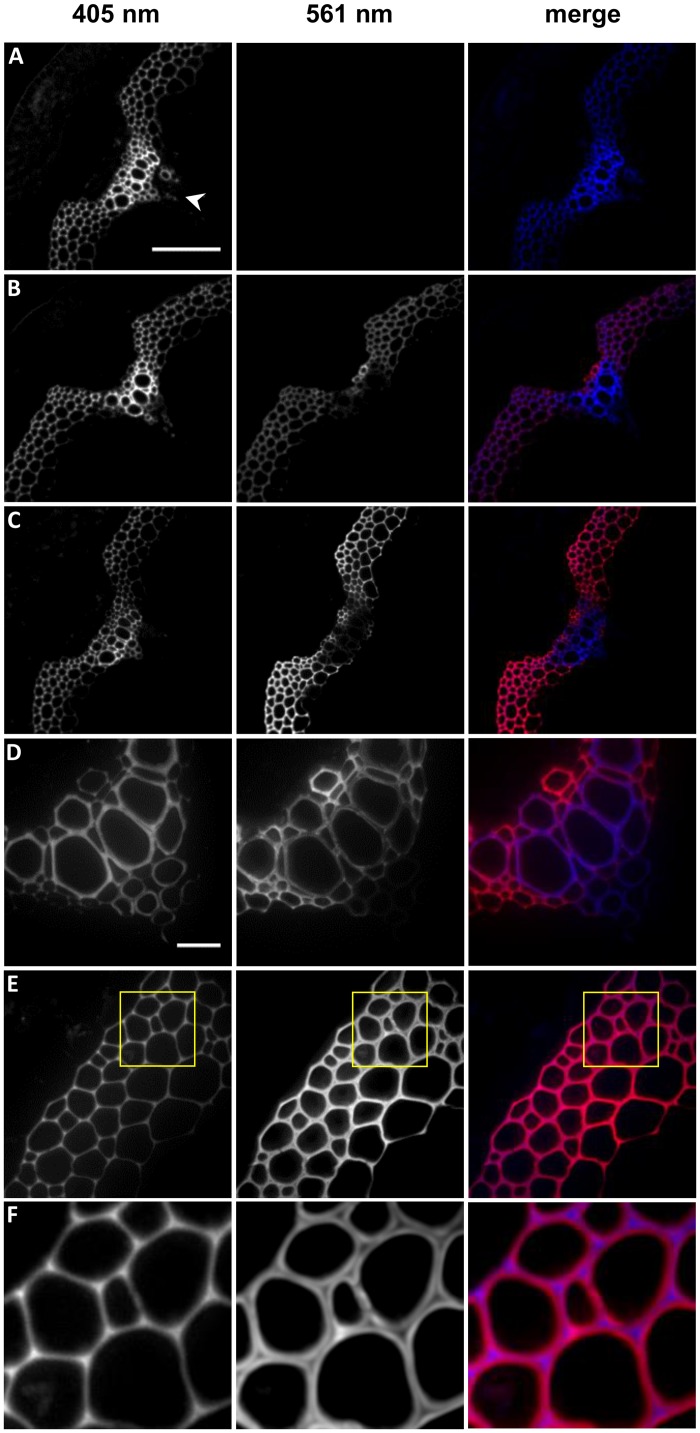
3-EPC incorporation in 40 μm-thick sections of 6 week old *Arabidopsis* stems. Autofluorescence (405 nm excitation) and click labeling (561 nm excitation) in (A) control section treated with 20 μM CA for 3 h, labeled with Alexa-594 azide for 1 h, and washed with 96% ethanol for 1 h; (B) section treated with 20 μM 3-EPC and 20 μM CA for 3 h, labeled with Alexa-594 azide for 1 h, and washed with 96% ethanol for 1 h; and (C) section treated with 20 μM 3-EPC for 3 h, labeled with Alexa-594 azide for 1 h, and washed with 96% ethanol for 1 h. (D) Xylem and (E) interfascicular fibers (IFFs) of section treated with 20 μM 3-EPC. (F) Zoomed-in region from image in (E), indicted by yellow box, showing incorporation patterns of 3-EPC in IFFs. Arrowhead in (A) indicates vascular bundle, with interfascicular fibers lying on either side. Images are contrast-enhanced maximum intensity projections of z series recorded with a spinning disk fluorescence confocal microscope. (A-C) were recorded using a 20X objective with a 561 nm laser at 15% power, 100 gain and 400 msec exposure time and a 405 nm laser at 100% power, 100 gain and 400 msec exposure time (scale bar, 100 μm). (D-E) were recorded using a 63X objective with a 561 nm laser at 15% power, 10 gain and 400 msec exposure time and a 405 nm laser at 100% power, 10 gain and 400 msec exposure time (scale bar, 20 μm).

To investigate whether the 3-EPC monolignol analog incorporates into naturally lignifying plant tissue, middle portions of stems from 6-week-old Arabidopsis Col-0 ecotype plants were sectioned using a cryostat and incubated with 3-EPC and/or CA for 3 h, then labeled with Alexa 594-azide for 1 h via a copper catalyzed click reaction. A 405 nm excitation laser was used to detect lignin autofluorescence and 561 nm excitation was used to detect click-associated fluorescence from Alexa 594-azide. Sections that were treated with 3-EPC (Fig 7B and 7C) showed significantly higher fluorescence with 561 nm excitation compared to sections treated with only CA and click labeling solution containing Alexa 594-azide, which showed no visible fluorescence (Fig 7A). Sections that were treated with a mixture of 20 μM 3-EPC and 20 μM CA (Fig 7B) showed lower fluorescence at 561 nm excitation after click labeling as compared to sections treated with 20 μM 3-EPC alone (Fig 7C), suggesting that CA might compete with 3-EPC at incorporation sites in the cell walls of stem sections, resulting in lower overall fluorescence. Imaging after 405 nm excitation of these sections revealed autofluorescence from both previously existing lignin and newly synthesized artificial lignin (Fig 7A–7C). Autofluorescence was higher in the vascular bundles or xylem (Fig 7D) than in interfascicular fibers (Fig 7E), whereas the click-associated 561 nm fluorescence was higher in the interfascicular fibers (Fig 7B–7D). Also, in the interfascicular fibers autofluorescence intensity was highest in the cell corners and middle lamellae and diminished inwards toward cell lumen, whereas click-associated fluorescence was not observed in the cell corners and had low intensity in the middle lamellae (Fig 7F). Lignin autofluorescence did not contribute to fluorescence in the 561 nm channel as observed in stem sections that were not treated with the 3-EPC or Alexa dye (S6 Fig). Quantification of autofluorescence (S8 Fig) revealed that there was no significant increase in autofluorescence after incorporation with 3-EPC and/or CA, which indicates that autofluorescence is mainly contributed by previously existing lignins. Therefore, fluorescence excitation at 405 nm and 561 nm allowed for differentiation between previously existing lignin and newly formed artificial lignin.

These results indicate that exogenously supplied monolignols and monolignol analogs can be incorporated into lignified cell walls of stem sections, with higher incorporation in regions with lower autofluorescence and relatively lower incorporation in regions with abundant previously existing lignins. Our observation that the newly synthesized artificial lignin was deposited in the secondary cell wall region after endogenous lignin was previously deposited in the cell corners and middle lamellae is in agreement with previous findings that lignin deposition begins in the cell corners and middle lamellae and then proceeds to the secondary cell wall [62–65]. In contrast to autofluorescence imaging, which cannot distinguish between existing and newly polymerized artificial lignin, the two-step process of incorporation and fluorescence labeling of 3-EPC allowed for specific detection of the locations of artificial lignification at what are possibly accessible lignin surface sites. The addition of CA reduced 3-EPC-associated labeling in stem sections (Fig 7B), in contrast with our results in seedlings, in which CA addition enhanced 3-EPC-associated labeling (Fig 6E). These results potentially reflect a difference between these tissues in that the stem sections contain large amounts of pre-existing lignins that might act as scaffolds onto which CA and 3-EPC competitively polymerize, whereas seedling root epidermal cells contain little if any pre-existing lignin so that CA polymerization could provide a scaffold for 3-EPC polymerization in artificial lignification reactions.

3-*O*-propargylcaffeyl alcohol (3-OPC), which is the click-compatible monolignol analog developed by Bukowski *et al*., also contains a modification at the 3’-C position of coniferyl alcohol, but includes a propargyl group in place of an ethynyl group [48]. In contrast to the observations reported here for 3-EPC, NMR analysis of DHP polymerized with 3-OPC revealed a clear presence of terminal alkyne groups and no novel linkages, indicating different polymerization characteristics for these two monolignol analogs. However, in plant tissues both 3-OPC and 3-EPC showed similar incorporation patterns in the epidermal layers of the *Arabidopsis* seedling roots as well as the lignifying tissues of the *Arabidopsis* stem.

In theory, after incorporation into plant tissue 3-EPC should be possible to image using Raman confocal microscopy without labeling with additional detection tags, because of the unique and strong Raman scattering signal at 2100 cm^-1^ exhibited by its ethynyl group. At this wavenumber, wall polysaccharides do not produce significant Raman scattering, making 3-EPC a potentially useful tool for specifically visualizing incorporated tagged monolignols [56,61]. Click chemistry-assisted fluorescent labeling of 3-EPC can also be potentially combined with super-resolution imaging techniques [66]. These attributes make 3-EPC a useful tag to study the dynamics of lignin polymerization in plant cell walls.

## Conclusions

In this study, we designed and tested a coniferyl alcohol analog, 3-EPC, as a probe to study lignin deposition. We demonstrated the incorporation of this monolignol tag, which has a small, bioorthogonal alkyne group modification on a natural lignin monomer, into *in vitro*-polymerized DHP lignin and plant cell walls. The versatility of this tag allows it to be labeled with different imaging probes like fluorophores or gold particles using a simple click chemistry reaction or to be detected without any chemical modifications using spectroscopic tools. This tag can be useful in identifying nucleation sites of lignification, especially if it can be successfully labeled with gold nanoparticles using click chemistry and detected using electron microscopy. Additionally, by providing for rapid detection of newly polymerized lignin, 3-EPC should enable analysis of the biochemical and architectural dependencies of the polymerization of exogenous monolignols in a large variety of plant tissues and cell types over different developmental stages. This and other tools [24,47,48] will allow for the unravelling of the molecular mysteries of lignin deposition.

## Supporting Information

S1 Fig NMR spectra of compound 2. ^1^H-NMR (A) and ^13^C-NMR (B) spectra of 4-hydroxy-3-iodobenzaldehyde, **2**.
(TIF)

S2 Fig NMR spectra of compound 3. ^1^H-NMR (A) and ^13^C-NMR (B) spectra of (E)-3-(4-hydroxy-3-iodophenyl)acrylic acid, **3.**
(TIF)

S3 Fig NMR spectra of compound 4. ^1^H-NMR (A) and ^13^C-NMR (B) spectra of (E)-methyl 3-(4-hydroxy-3-iodophenyl)acrylate, **4.**
(TIF)

S4 Fig NMR spectra of compound 5. ^1^H-NMR (A) and ^13^C-NMR (B) spectra of (E)-methyl 3-(4-hydroxy-3-((trimethylsilyl)ethynyl)phenyl)acrylate, **5.**
(TIF)

S5 Fig NMR spectra of 3-EPC, 6. ^1^H-NMR (A) and ^13^C-NMR (B) spectra of 3-ethynyl *p-*coumaryl alcohol (3-EPC), **6.**
(TIF)

S6 Fig Fluorescence in untreated 40 m thick sections of lignified 6-week-old *Arabidopsis* stems. Autofluorescence was measured with 405 nm excitation and click labeling with 561 nm excitation. Images are contrast-enhanced maximum intensity projections of z series recorded with a spinning disk fluorescence confocal microscope using a 20X objective. Images were recorded using a 561 nm laser at 15% power, 100 gain and 400 msec exposure time and a 405 nm laser at 100% power, 100 gain and 400 msec exposure time (Scale bar, 100 μm).
(TIF)

S7 Fig Quantification of fluorescence intensities in different developmental zones of seedling roots. Quantified click labeling-associated fluorescence (561 nm excitation) in the early elongation zone (EEZ), late elongation zone (LEZ), early differentiation zone (EDZ), and late differentiation zone (LDZ) of seedlings treated with 20 μM 3-EPC, 5 μM 3-EPC + 15 μM CA, and 20 μM CA. Data were averaged from 9 samples per treatment; error bars indicate standard error. Numbers indicate statistically different data sets (P<0.05, t-test) within each treatment group, and letters with different symbols (none, ‘, or *) indicate statistically different data sets (P<0.05, t-test) within each root zone group (EEZ: a; LEZ: b; EDZ: c; LDZ: d).
(TIF)

S8 Fig Quantification of fluorescence intensities per unit area in stem sections. Quantified click labeling-associated fluorescence (561 nm excitation) and autofluorescence (405 nm excitation) in sections treated with 20 μM 3-EPC, 20 μM 3-EPC + 20 μM CA, and 20 μM CA and untreated sections. Data were averaged from 9 samples per treatment; error bars indicate standard error. Numbers indicate statistically different data sets (P<0.05, t-test) for 561 nm excitation and letters indicate statistically different data sets (P<0.05, t-test) for 405 nm excitation.
(TIF)
