# Supplementary figures and images for: A Versatile Click-Compatible Monolignol Probe to Study Lignin Deposition in Plant Cell Walls
Source: PLoS One. 2015 Apr 17;10(4):e0121334. doi: 10.1371/journal.pone.0121334 (PMC4401456; doi:10.1371/journal.pone.0121334)

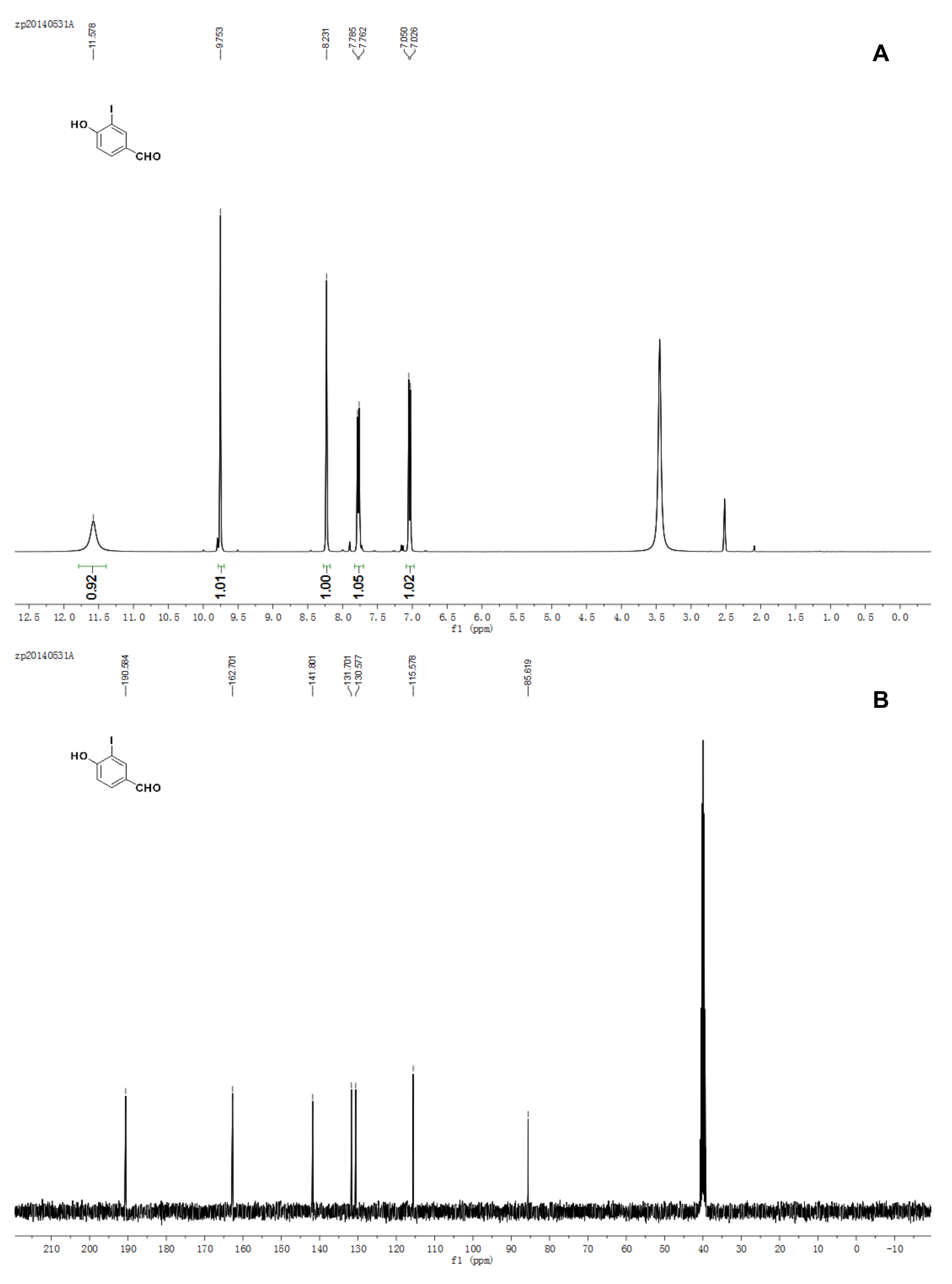

Supplement: S1 Fig — 1H-NMR (A) and 13C-NMR (B) spectra of 4-hydroxy-3-iodobenzaldehyde, 2. (TIF) [file pone.0121334.s001.tif]

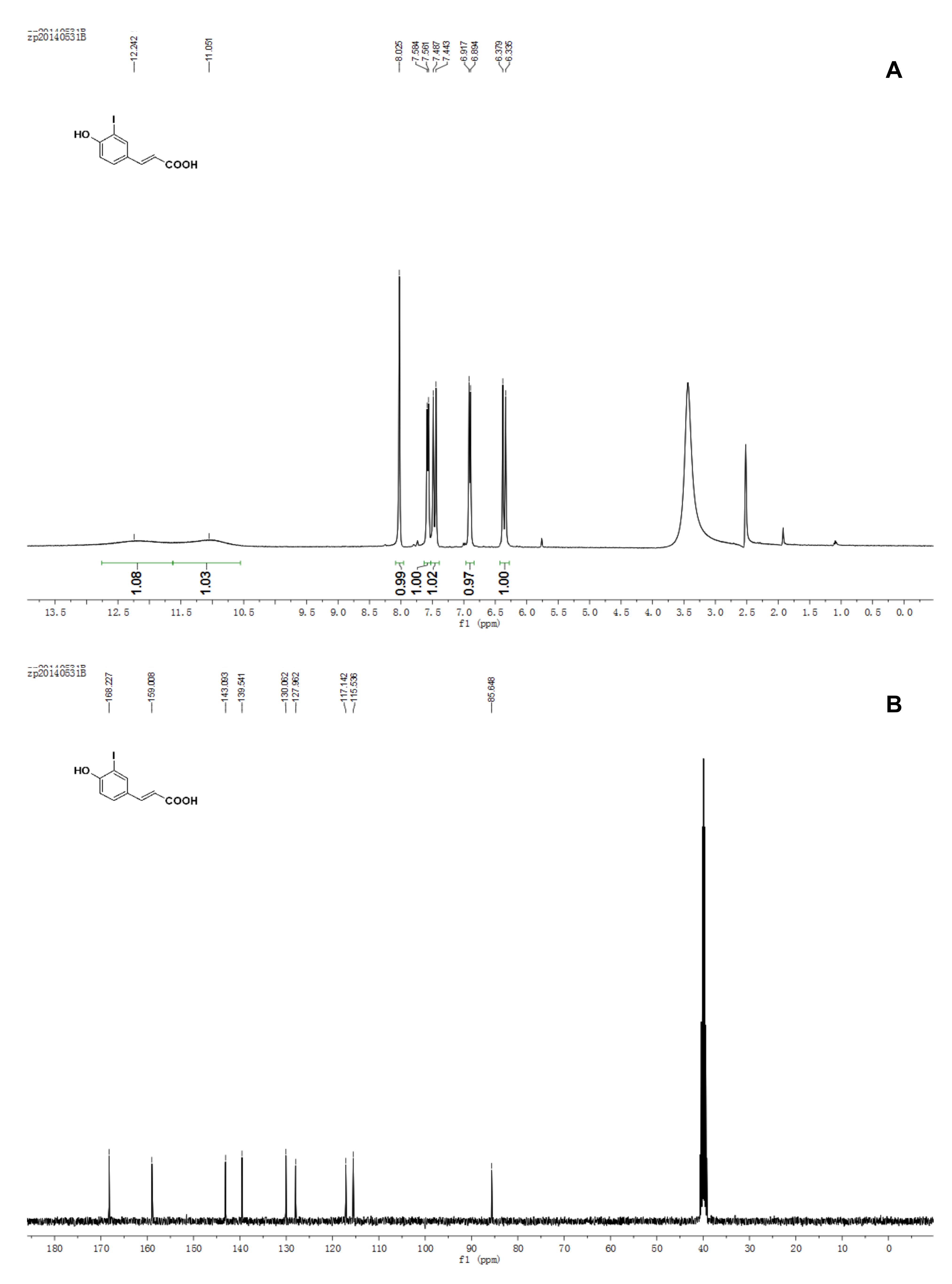

Supplement: S2 Fig — 1H-NMR (A) and 13C-NMR (B) spectra of (E)-3-(4-hydroxy-3-iodophenyl)acrylic acid, 3. (TIF) [file pone.0121334.s002.tif]

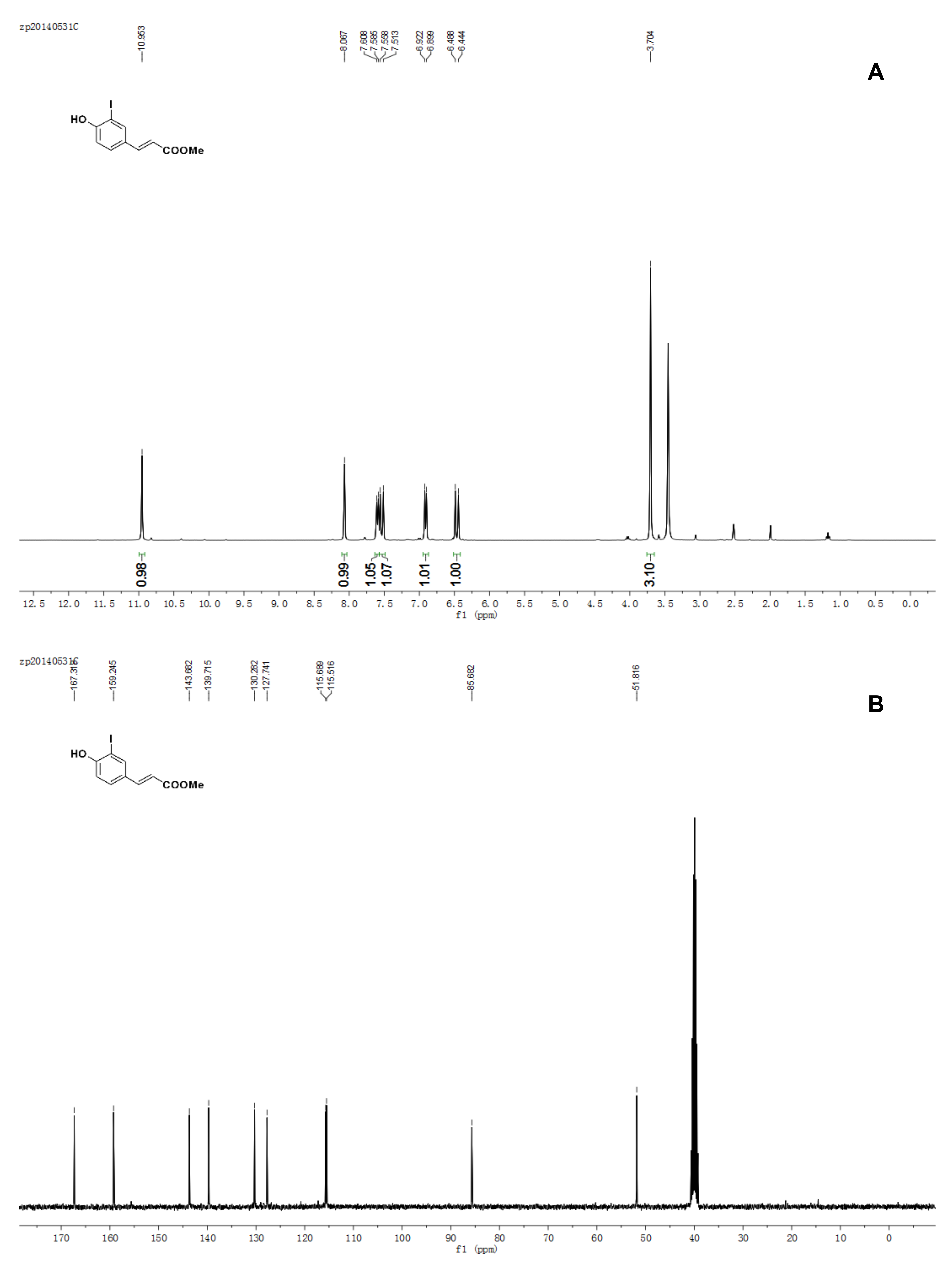

Supplement: S3 Fig — 1H-NMR (A) and 13C-NMR (B) spectra of (E)-methyl 3-(4-hydroxy-3-iodophenyl)acrylate, 4. (TIF) [file pone.0121334.s003.tif]

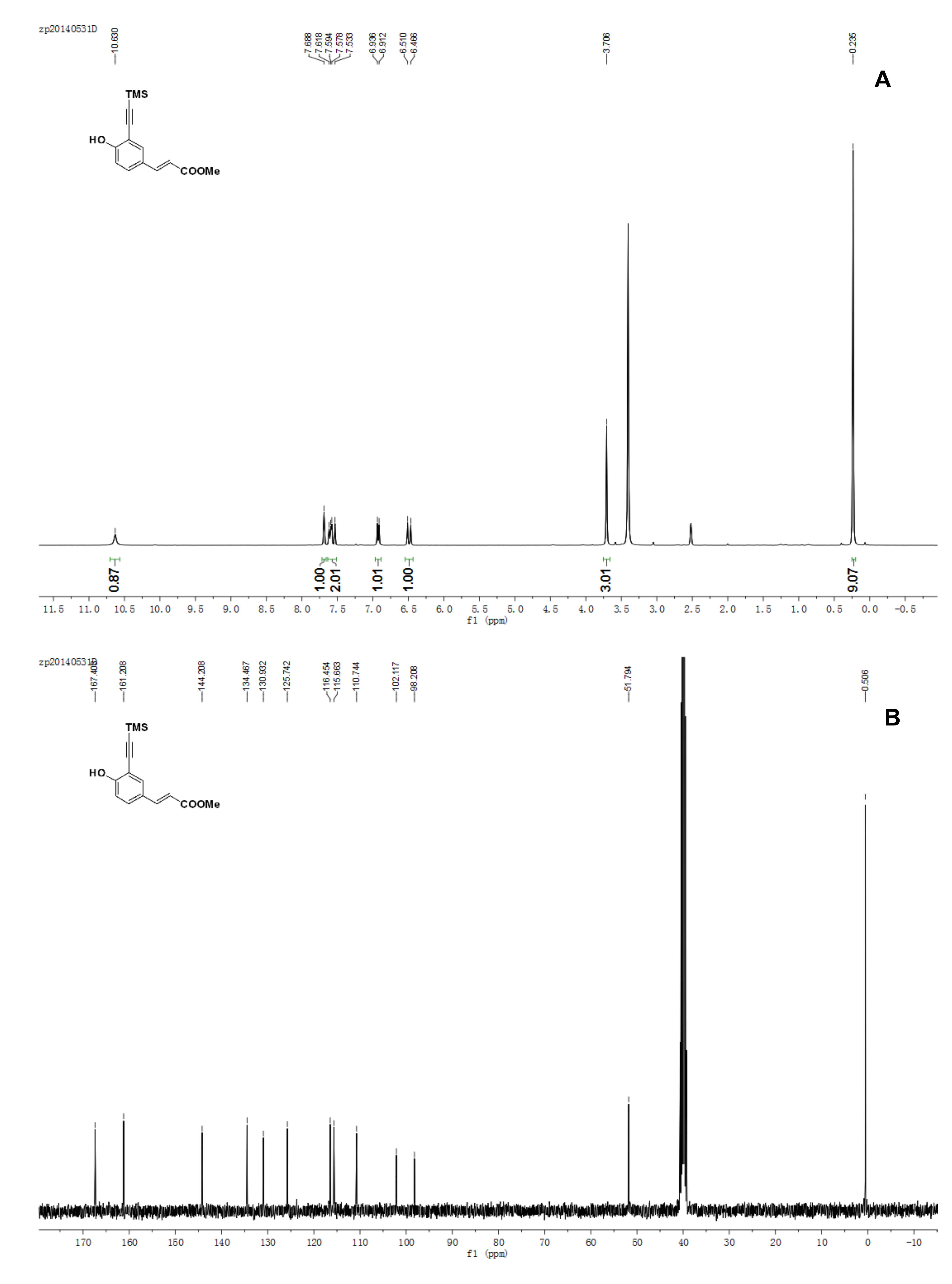

Supplement: S4 Fig — 1H-NMR (A) and 13C-NMR (B) spectra of (E)-methyl 3-(4-hydroxy-3-((trimethylsilyl)ethynyl)phenyl)acrylate, 5. (TIF) [file pone.0121334.s004.tif]

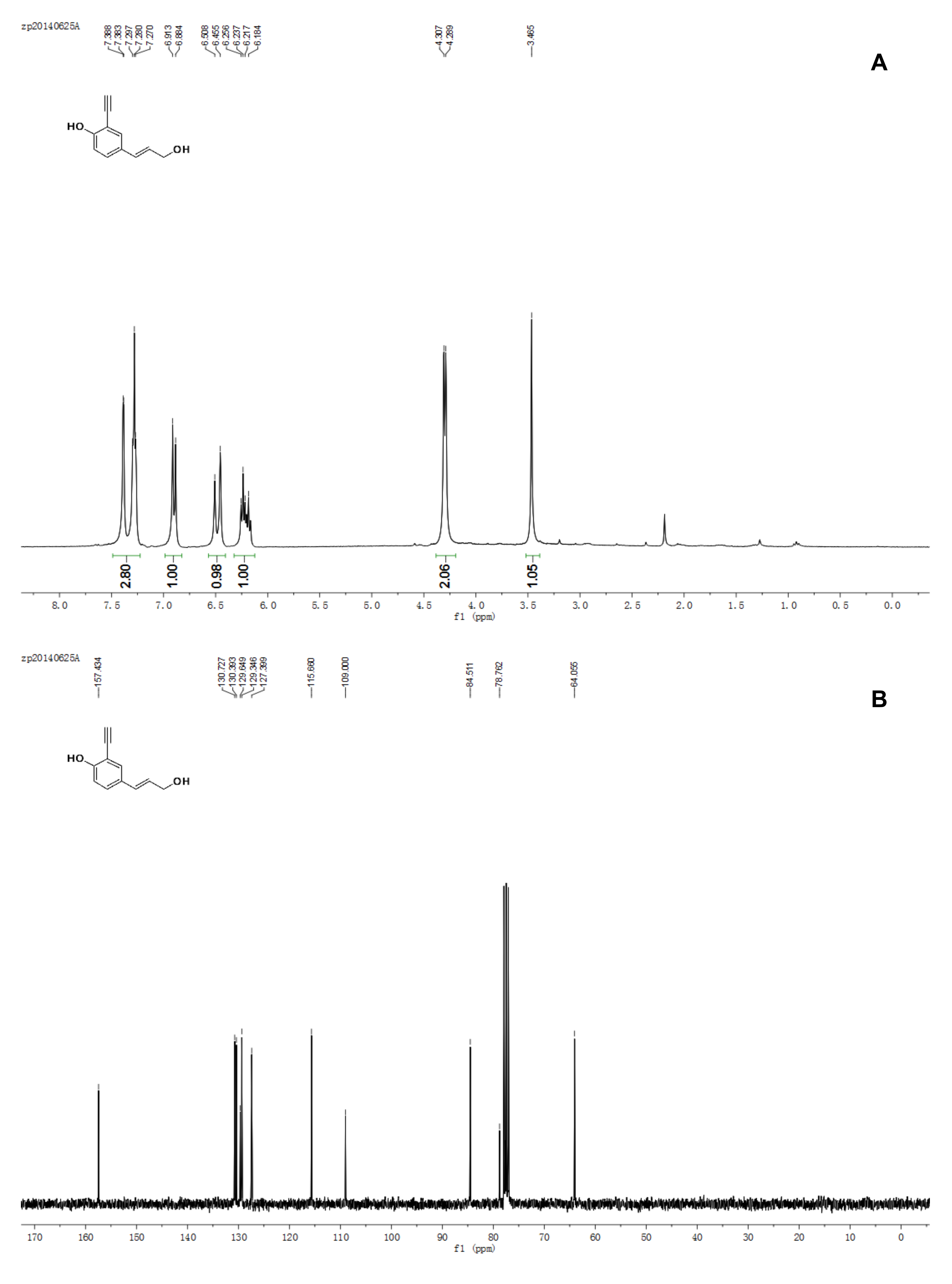

Supplement: S5 Fig — 1H-NMR (A) and 13C-NMR (B) spectra of 3-ethynyl p-coumaryl alcohol (3-EPC), 6. (TIF) [file pone.0121334.s005.tif]

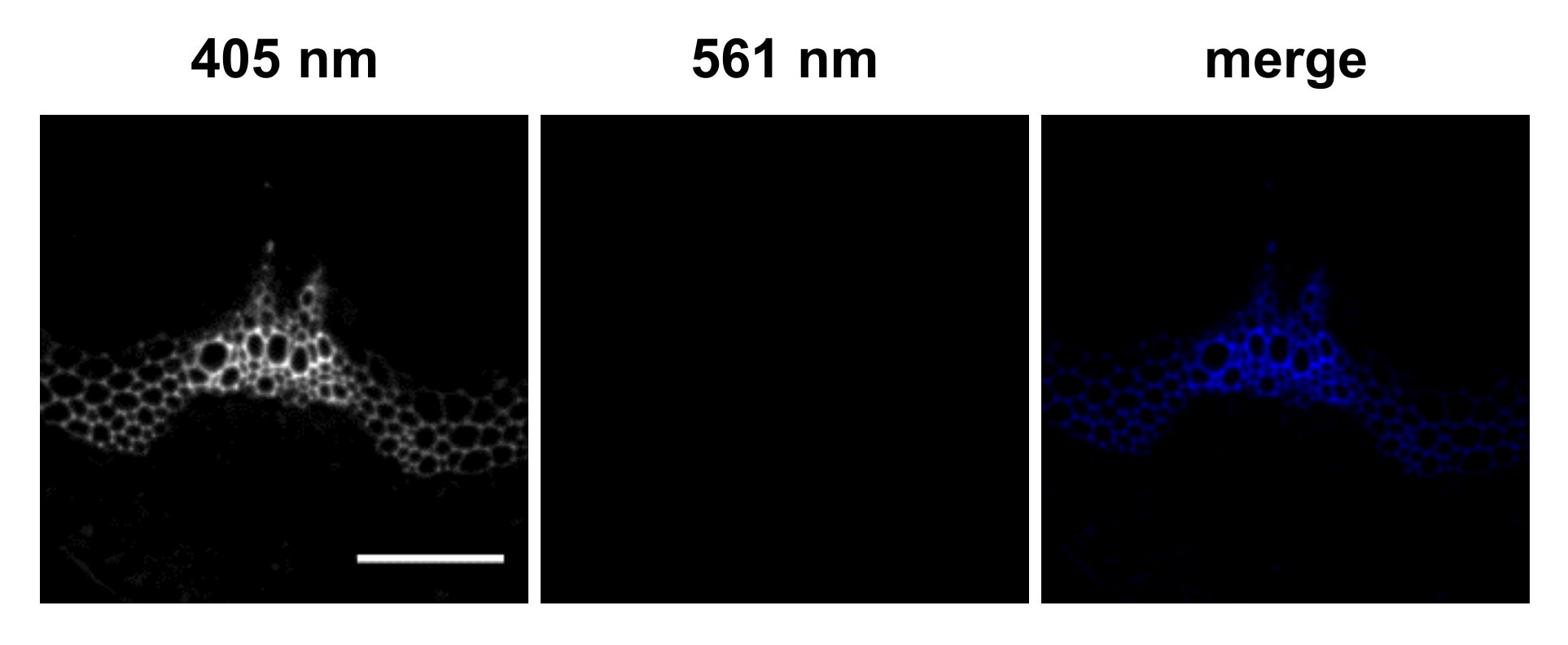

Supplement: S6 Fig — Autofluorescence was measured with 405 nm excitation and click labeling with 561 nm excitation. Images are contrast-enhanced maximum intensity projections of z series recorded with a spinning disk fluorescence confocal microscope using a 20X objective. Images were recorded using a 561 nm laser at 15% power, 100 gain and 400 msec exposure time and a 405 nm laser at 100% power, 100 gain and 400 msec exposure time (Scale bar, 100 μm). (TIF) [file pone.0121334.s006.tif]

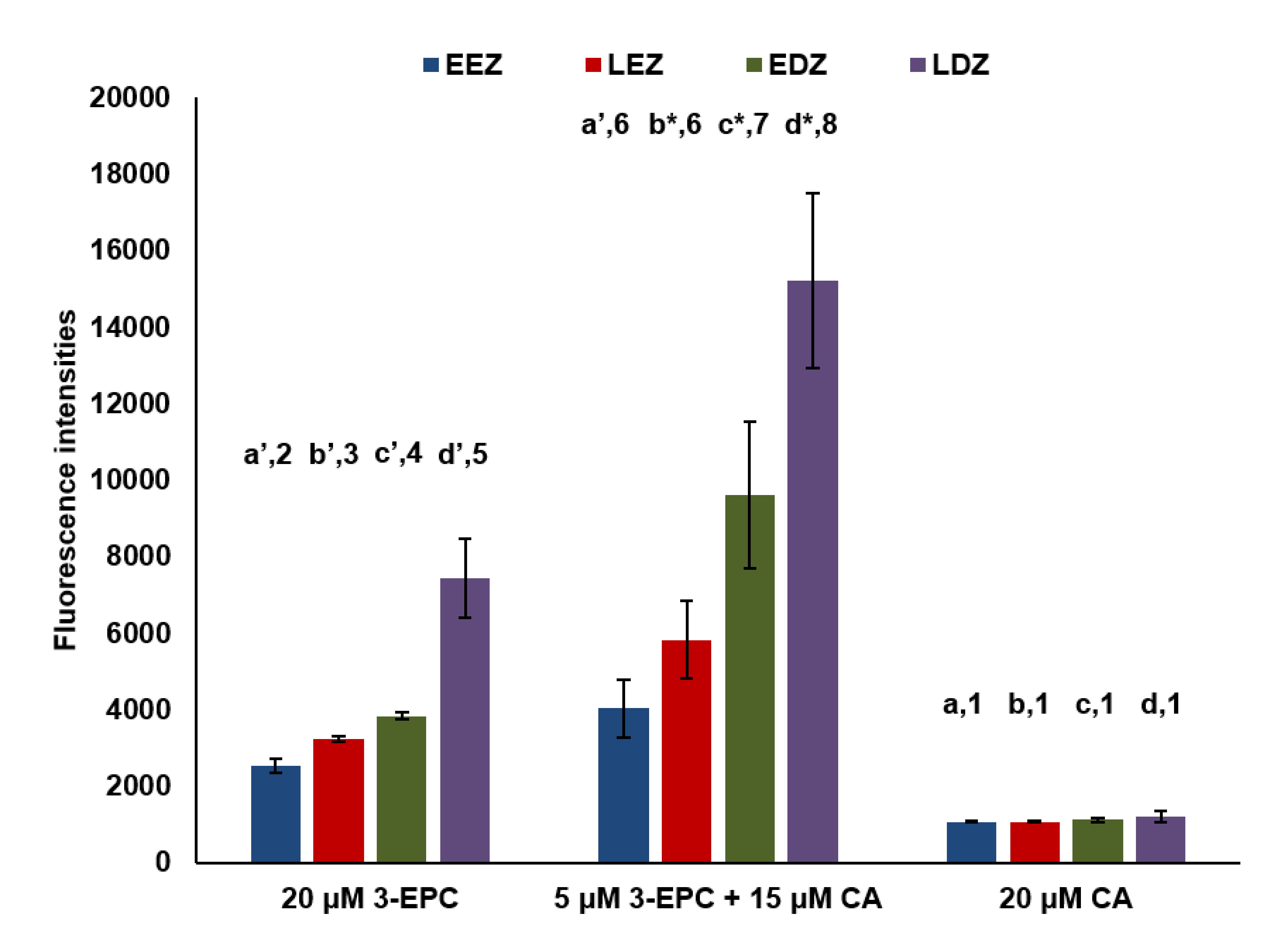

Supplement: S7 Fig — Quantified click labeling-associated fluorescence (561 nm excitation) in the early elongation zone (EEZ), late elongation zone (LEZ), early differentiation zone (EDZ), and late differentiation zone (LDZ) of seedlings treated with 20 μM 3-EPC, 5 μM 3-EPC + 15 μM CA, and 20 μM CA. Data were averaged from 9 samples per treatment; error bars indicate standard error. Numbers indicate statistically different data sets (P<0.05, t-test) within each treatment group, and letters with different symbols (none, ‘, or *) indicate statistically different data sets (P<0.05, t-test) within each root zone group (EEZ: a; LEZ: b; EDZ: c; LDZ: d). (TIF) [file pone.0121334.s007.tif]

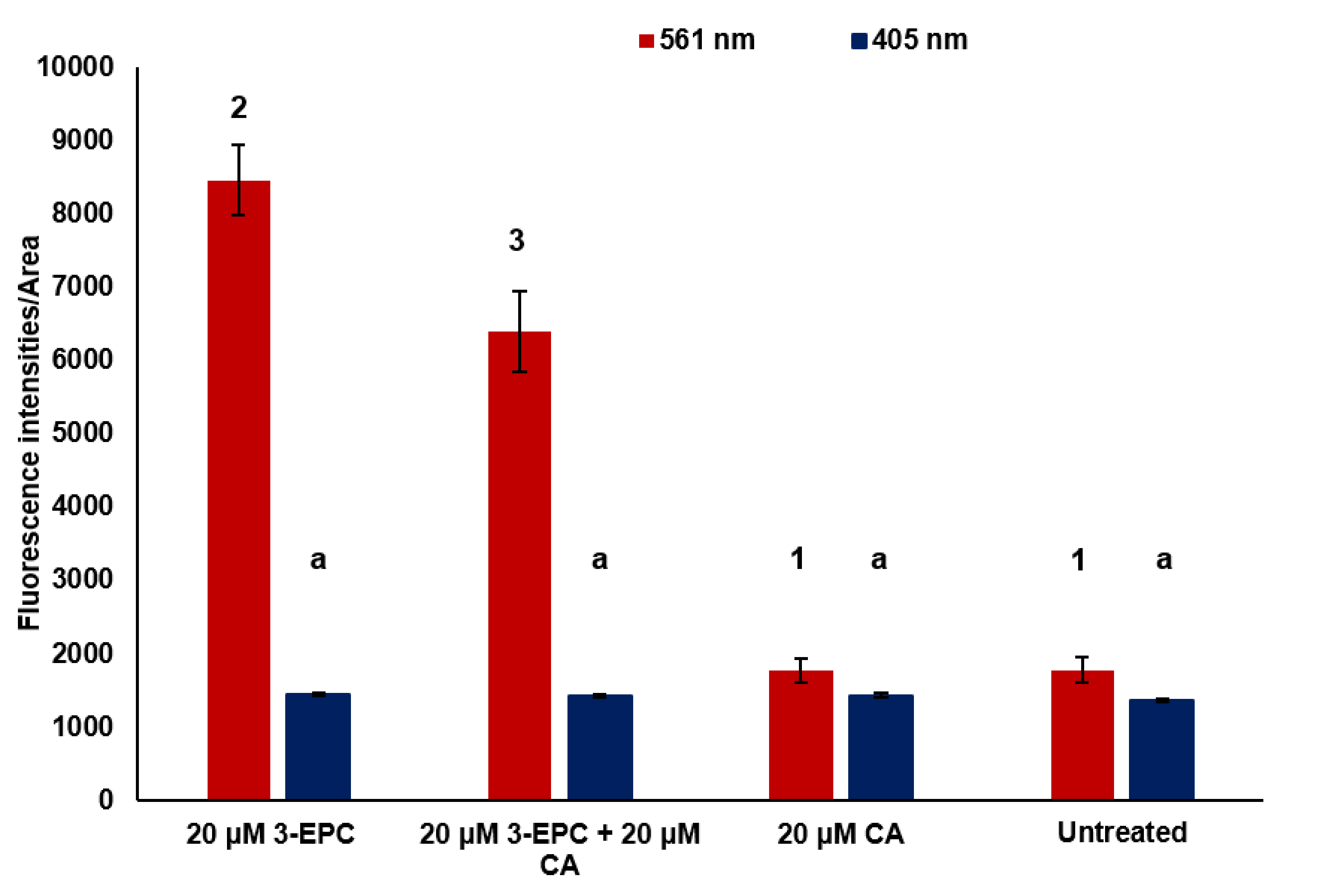

Supplement: S8 Fig — Quantified click labeling-associated fluorescence (561 nm excitation) and autofluorescence (405 nm excitation) in sections treated with 20 μM 3-EPC, 20 μM 3-EPC + 20 μM CA, and 20 μM CA and untreated sections. Data were averaged from 9 samples per treatment; error bars indicate standard error. Numbers indicate statistically different data sets (P<0.05, t-test) for 561 nm excitation and letters indicate statistically different data sets (P<0.05, t-test) for 405 nm excitation. (TIF) [file pone.0121334.s008.tif]
